# The potential key role of choroidal non-perfusion and rod degeneration in the pathogenesis of macular neovascularization type 3

**DOI:** 10.1038/s41433-024-03034-z

**Published:** 2024-03-18

**Authors:** Bilal Haj Najeeb, Ursula Schmidt-Erfurth

**Affiliations:** Department of Ophthalmology and Optometry, Medical University of Vienna, Vienna, Austria

**Keywords:** Macular degeneration, Pathogenesis

## Abstract

Macular neovascularization type 3 (MNV3) is a multifactorial disease with distinct epidemiological, clinical, pathomorphological and topographical characteristics. This review of the literature discusses the latest experimental and clinical outcomes that could explain the pathogenesis of retinal neovascularization. Although patients with MNV3 are usually older than those with MNV1 or 2, their lesions do not coexist with, precede, or follow other types in the same eye. The regional distribution of MNV3 lesions is characterized as confined to the parafoveal macula without any involvement of the rod-free foveal area. Focal outer retinal atrophy and choroidal non-perfusion are the main structural features that occur prior to the development of retinal neovascularization. Also, histological and experimental studies of MNV3 and other non-neovascular age-related macular degeneration diseases complicated with MNV3-like lesions strongly suggest rod degeneration contributes to the pathogenesis. Therefore, the retinal neovascularization in MNV3 has a different pathogenesis from the choroidal neovascularization in MNV1 and 2 and emerging evidence indicates that choroidal non-prefusion and rod degeneration play a key role in the pathogenesis of MNV3. Accordingly, we suggest a sequence of pathological events that start with choroidal non-perfusion due to advanced age followed by hypoxia of the outer retina at the parafoveal area. This induces a remarkable degeneration of rods that triggers the growth of retinal neovascularization due to the imbalance of the angiogenic factors in the outer retina.

## Abstract (zh)

3型黄斑新生血管 (MNV3) 具有独特流行病学、临床、病理形态和地形特征的多因素疾病。本文回顾讨论了一些最新的实验和临床结果, 可以解释视网膜新生血管的发病机制。尽管MNV3的患者通常比MNV1或2的患者年纪大, 但病变不会在同一眼发生的其他NV类型共存、先于或伴随其他类型。MNV3病变的区域分布特点是局限于旁黄斑中央凹, 不累及无视杆细胞的黄斑中心区域。局部外层视网膜萎缩和脉络膜无灌注是视网膜新生血管前期的主要病理改变特征。此外, MNV3和其他复杂的、带有MNV3样病变的nAMD的组织学和实验研究表明视杆细胞退行性变可能促进了其的发生与发展。因此, MNV3中的视网膜新生血管与MNV1及MNV2中的CNV有不同的发病机制, 新的证据表明脉络膜无灌注和视杆细胞退行性变在MNV3的发病机制中起关键作用。据此, 我们提出一系列病理发病机制的假说, 即由于年龄老化导致的脉络膜无灌注, 导致脉络膜旁外层视网膜缺氧。由于外层视网膜的血管生成因子表达失衡, 诱导了视杆细胞的显著退化, 从而触发了视网膜新生血管的增长。

## Introduction

Macular neovascularization type 3 (MNV3), earlier known as retinal angiomatous proliferation, is a distinct form of neovascular age-related macular degeneration (nAMD) that is characterized by severe angiogenic activity and poor prognosis and is associated with subretinal drusenoid deposits (SDD) [1–7]. It is more prevalent in the Western and Mediterranean regions, which could be explained by higher life expectancy and non-Asian ethnicity. The etiology of MNV3 is still poorly understood. Recently, modern imaging techniques have added morphological and topographical insights which have expanded our understanding on the pathogenesis of this type of neovascularization. It has become possible to detect it at the precursor stage by observing migrated retinal pigment epithelium (RPE) cells in the outer retina [8]. These cells have been suggested to trigger retinal angiogenesis [9]. However, they are not specific to MNV3 as they also occur in non-exudative macular degeneration [10]. Furthermore, their number in eyes with MNV3 outweighs the number of retinal neovascularizations [11]. Other structural and vascular changes, such as reduced choroidal perfusion, have also been presumed to be crucial factors [12]. Also, our analyses of the regional distribution of the lesions in the macula suggested a causal relation between rod degeneration and the development of MNV3 lesions [13]. Interestingly, MNV3 in eyes, even in their multifocal phenotype, is not preceded by MNV1 or MNV2 nor does it coexisted with these lesions, even though patients with MNV3 are usually older than those with the other types [2, 13–15]. Thus, eyes with retinal neovascularization in MNV3 definitively have a pathological pathway different from choroidal neovascularization seen in MNV1 and 2. Accordingly, doubt has been raised about the benefit gained from AREDS 2 supplements in MNV3 [7].

Few histological studies have been carried out on human donor eyes and animal models to date. Here, we draw on robust basic and clinical research outcomes to review the main pathological course of MNV3, unravel any potential causal relations and describe the possible sequence of events leading to its development.

## Ageing

Ageing is the primary non-modifiable risk factor of AMD. The influence of ageing is particularly prominent in MNV3 [16]. Similarly, SDD affect advanced-aged patients with AMD [17]. Rods are also more susceptible to degenerate with age, while cones and RPE remain resilient due to additional support from Müller cells and the proximity to choriocapillaris, respectively [18]. These findings indicate that MNV3 and rods are linked and are preferentially influenced by advanced age. This relation can be appreciated through a recent study that revealed focal atrophy involving the photoreceptor layer at sites prior to the development of MNV3 lesions [19].

## Topography

Rods are the predominant photoreceptors in the retinal tissue, even in the macula which contains the highest density of cones [20]. Interestingly, almost all MNV3 lesions occur in the parafoveal area (500–1500 µm from the foveal center), where rods show the greatest age-induced loss [13, 14, 20]. Moreover, other topographical studies have reported that the outer macula, where rods are highly concentrated, is coincident with the area of the highest distribution of SDD, which are considered a specific risk factor for MNV3 [3, 18].

Delayed rod-mediated dark adaptation (RMDA) is generally considered a novel functional biomarker of AMD [21]. It is observed some years prior to the evolution of early AMD without any associated change in the performance of cone-mediated tasks such as photopic acuity, contrast and light sensitivity [22]. A recent study found that RMDA in eyes with early or intermediate AMD and SDD was significantly more delayed in the parafoveal area compared with the perifoveal area (>1500 µm from the foveal center) [23]. In addition, MNV3 is the only type of MNV that does not occur in the foveal area, which is histologically devoid of rods, even when the retinal foveal avascular zone is extremely small [13]. Furthermore, drusen, which are the hallmark of AMD, follow the distribution of cone density in the foveal area, where mainly MNV1 and 2 lesions occur. However, SDD, which indicate progressive photoreceptor damage, are distributed more in the extrafoveal area where MNV3 develop [24, 25]. These outcomes indicate that MNV3, unlike MNV1 and 2, has a strong topographic relation with rod damage, and SDD is a relevant biomarker for both pathologies (MNV3 and rod degeneration).

## Choriocapillaris perfusion

**Fig. 1:**
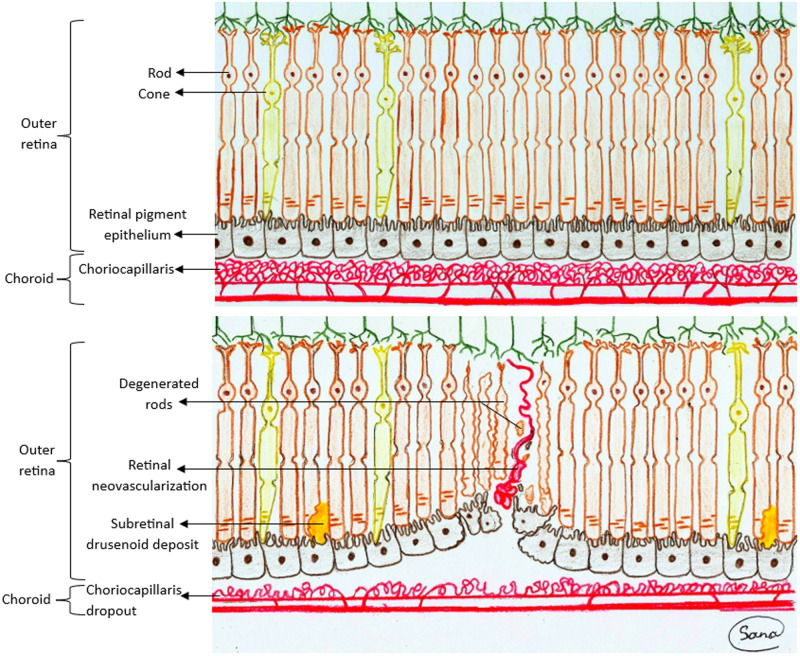
A schematic diagram of the early changes of macular neovascularization type 3 (MNV3). The upper image shows the normal retinal and choroidal structures. The lower image illustrates the early pathomorphological changes of MNV3. Note the primary remarkable thinning of the choroid and the decrease of the choroidal blood supply, in particular the choriocapillaris layer. This causes degeneration of rods that is manifested as thinning of the outer retinal layer and formation of subretinal drusenoid deposits. Consequently, retinal neovessels originating from the deep capillary plexus extend to the subretinal space through the degenerated neurosensory retina and retinal pigment epithelium.

The first structural change seen in aged healthy macula is a decrease in choriocapillaris density [26]. Interestingly, eyes with MNV3 present with significant choroidal thinning using optical coherence tomography (OCT) even prior to the onset of neovascularization, compared with age-matched healthy eyes or eyes with other MNV types [27]. In addition, OCT angiography studies did not detect any retinal non-perfusion, but only perfusion deficit in the choriocapillaris layer [12] indicating a decrease in the supply of oxygen and nutrients to the outer retina [28]. Similarly, SDD, which usually increase with age, are found over thinned choroid and are accompanied by a further decease of dark adaptation, indicating a concurrent rod degeneration [20, 29–31]. Moreover, a multimodal imaging analysis revealed that SDD are colocalized with areas of greatest choroidal ischemia (choroidal watershed zones) [32, 33]. Likewise, MNV3 lesions tend to distribute along radial bands extending from the perifoveal zone and overlay the area of greatest age-related rod loss [13]. Furthermore, the predilection of MNV3 lesions to occur in the temporal half of the macula could be explained by the fewer number of posterior ciliary arteries supplying each choroidal segment in the temporal macular half compared with the nasal one [13, 34]. Also, the inadvertent extensive exudation of MNV3 lesions underwent photodynamic therapy (PDT) support the role of choroidal non-perfusion in the pathogenesis of this type, as choriocapillaris occlusion followed by choroidal thinning is a known complication of PDT [35, 36]. These findings support a tight relation of perfusion deficiency of the choriocapillaris with both the degeneration of rods and development of MNV3 (Fig. 1).

## Diseases characterized with primary rod loss

Enhanced S cone dystrophy is a rare autosomal recessive disease caused by a gene mutation of a specific nuclear receptor responsible for determining the photoreceptor phenotype in the outer retina [37]. Different variants of gene mutations that lead to a unique change in photoreceptors differentiation have been discovered. Histologically, a severe degeneration to complete loss of rods is seen, together with approximately double the number of cones, the majority of which are blue cones, and a decrease in red/green cones [38]. The main initial symptom is nyctalopia, manifested clinically as a very weak to undetectable signal of rod-specific dark adaptation. Other clinical signs are central or peripheral retinoschisis, yellow-white dots and nummular pigmentation. Most importantly, in a multimodal imaging study of 93 patients, 14 (15%) demonstrated retinal angiogenic lesions similar to type 3 ones. The lesions in this progressive disease affect very young patients (2 years of age) as well as old individuals [39]. Notably, no lesions compatible to MNV1 or 2 were observed [39]. Likewise, all recent case reports of retinitis pigmentosa using OCT angiography identified only MNV3-like lesions as a sequela [40, 41]. This distinguishing retinal neovascularization developed after a primary severe loss of rods reinforces the hypothesis that rod degeneration is an indispensable event for the development of MNV3 lesions [13].

## Experimental and histological studies

Recent studies using animal models have shown that avascular outer retinas can be invaded by retinal neovessels when certain genes of either or both photoreceptors are knocked out. They showed a subsequent deficiency of specific locally secreted anti-angiogenic factors from the degenerated photoreceptors. Of course, the contribution of rods to this change is supposed to be more than that of cones, as they are the most numerous and vulnerable photoreceptors (the rods to cones ratio is 20:1 in the periphery and 9:1 in the macular region) [42–44]. These experimental outcomes imply that degenerated rods can stimulate the growth of MNV3-like lesions.

Moreover, retinal neovascularization has been noted in other hypoxic experiments simulating the impairment of choroidal perfusion [45]. Also, histological studies on human donor eyes have shown diffuse loss or sclerosis of the choriocapillaris and massive photoreceptor degeneration very close to the retinal neovascularization [15, 46, 47]. A focal thinning of the outer retina, suggesting photoreceptor damage even prior to the development of neovessels, was documented using OCT [8, 19].

On account of the above-mentioned robust findings, we believe that in patients with MNV3, who are typically older than those with other types, the damage to rods is amplified and promoted due to underlying choroidal non-perfusion. Thus, both pathologies play a key role in the development of retinal neovascularization. Accordingly, we hypothesize the following cascade of events (Fig. 1):

Advanced age promotes a decrease in choroidal perfusion. This induces hypoxia in the outer retina, which is greatest along the watershed zones, that preferentially causes degeneration of rods. Subsequently, rod damage induces an imbalance of the anti-angiogenic status in the outer retina ending up with development of retinal neovascularization.

## Conclusion

MNV3 is a particularly age-related disease. Recent clinical, histological and experimental findings have helped us to better understand the pathomechanism of the disease. Although its etiology is multifactorial, evidence suggests that choroidal non-perfusion and rod degeneration are major factors.
